# Sleep Promotion Program for Improving Sleep Behaviors in Adolescents: A Randomized Controlled Pilot Study

**DOI:** 10.1155/2016/8013431

**Published:** 2016-03-20

**Authors:** Bindu John, Sumanth Shetty Bellipady, Shrinivasa Undaru Bhat

**Affiliations:** ^1^Nursing Department, College of Health Sciences, University of Bahrain, P.O. Box. 32038, Sakheer, Bahrain; ^2^Department of Pediatrics, K S Hegde Medical Academy, Mangalore 575 018, India; ^3^K S Hegde Medical Academy, Mangalore 575 018, India

## Abstract

*Aims*. The purpose of this pilot trial was to determine the efficacy of sleep promotion program to adapt it for the use of adolescents studying in various schools of Mangalore, India, and evaluate the feasibility issues before conducting a randomized controlled trial in a larger sample of adolescents.* Methods*. A randomized controlled trial design with stratified random sampling method was used. Fifty-eight adolescents were selected (mean age: 14.02 ± 2.15 years; intervention group, *n* = 34; control group, *n* = 24). Self-report questionnaires, including sociodemographic questionnaire with some additional questions on sleep and activities, Sleep Hygiene Index, Pittsburgh Sleep Quality Index, The Cleveland Adolescent Sleepiness Questionnaire, and PedsQL*™* Present Functioning Visual Analogue Scale, were used.* Results*. Insufficient weekday-weekend sleep duration with increasing age of adolescents was observed. The program revealed a significant effect in the experimental group over the control group in overall sleep quality, sleep onset latency, sleep duration, daytime sleepiness, and emotional and overall distress. No significant effect was observed in sleep hygiene and other sleep parameters. All target variables showed significant correlations with each other.* Conclusion*. The intervention holds a promise for improving the sleep behaviors in healthy adolescents. However, the effect of the sleep promotion program treatment has yet to be proven through a future research. This trial is registered with ISRCTN13083118.

## 1. Introduction

The adolescent period is the most vulnerable period for sleep problems, and adolescents in the age group of 12–17 years are one of the most sleep deprived age groups in the society [1]. Sleep is essential for adolescents for the physical health, as well as for cognitive and affective functioning [2]. The prefrontal cortex, which governs abstract thinking, creativity, and the tasks involved with higher order neurocognitive functioning, is known to be sensitive to sleep deprivation [3]. Research shows that adolescents require 9 hours of average sleep per night [4], but social and educational demands compel them to sleep less [5].

Most studies on adolescent sleep patterns show that the bedtime and the sleep duration decrease with increasing age, which results in a shorter night time sleep [2, 6, 7]. This deficit leads to increased daytime sleepiness and affects the daytime functioning in adolescents [8]. A decrease in total sleep time increasing with higher grades is also reported in studies done in India [9], Japan [10], Taiwan [11], and the USA [12]. Poor sleep, increased sleep fragmentation, and later bedtimes with consistent wake-up times can seriously affect learning capacity and school performance [3] in adolescents, as well as triggering higher negative moods such as anger, confusion, depression, fatigue, and tension [1]. In a study among 4175 adolescents aged 11–17 years at baseline and a subsequent follow-up in 3134 adolescents in Houston, Texas, the results added to the growing evidence that the burden of insomnia among adolescents is comparable to that of other major psychiatric disorders such as mood and anxiety disorders, depressive disorders, and substance abuse [13]. Insufficient sleep, arising from the biological, psychological, sociocultural [14], lifestyle, and environmental factors, may also have a significant negative impact on adolescents functioning at home and in the academic settings [15].

Many sleep difficulties were reported in earlier research conducted in adolescents. In a study conducted in Italy among 339 high school students, complaints of bad sleep (19%) and sleep fragmentation with trouble falling asleep (45%) were found to be common [16]. In another study, insufficient sleep was reported by one-fourth to one-fifth of adolescents and only 17.2% of the youth reported sleeping for an optimal amount of 9 hours, 28.8% reported sleeping 8 hours on weeknights, and over 43% slept 7 hours or less on weeknights. The negative outcomes associated with insufficient sleep range from sleepiness and mood disturbances to lack of attention, poor grades, behavioral problems, depression, and overweight [17, 18]. In an Australian based randomized controlled trial study on 81 students, 95% of the subjects at baseline reported at least one type of sleep problem [19]. Other health-related complaints reported in studies among adolescents were daytime leg pain [9], feeling unrefreshed/tired in the morning [20], difficulties falling asleep [7], back pain and shoulder pain, headache, emotional problems like feelings of sadness, irritability, and nervousness [21], and daytime sleepiness [3, 7, 20]. Daytime sleepiness was also found to lower the school performance in adolescents [20, 22].

Sleep hygiene is the practice of several behaviors that optimize good sleep and improve daytime functioning [23] and can alleviate behavioral sleep problems, which are believed to be caused by behavioral factors and could otherwise lead to sleep disruption and daytime sleepiness in adolescents [24]. Inadequate sleep hygiene, such as practices that produce increased arousal and interfere with the sleep organization, can be modified through numerous behavioral and environmental practices like adhering to consistent bedtimes and rise times, minimize napping during the day, having a relaxing sleep time schedule, avoiding caffeinated beverages or stimulants four hours prior to bedtime, and encouraging a favorable sleeping environment [25, 26]. Sleep hygiene is found to be significantly correlated with good perceived sleep quality, and sleep hygiene along with home atmosphere explained 22% variance in perceived sleep quality [27, 28]. It is possible that the subjects who have poor sleep quality (e.g., sleep onset difficulty or sleep fragmentation) could subsequently develop inadequate sleep habits, including an irregular sleep schedule, and also there are myriads of inadequate sleep habits and sleep-related behaviors likely to make the sleep quality vulnerable in normal subjects [29]. Daytime sleepiness, which is mostly a consequence of insufficient sleep, is found to have an inverse association with total night time sleep duration [22, 30] as well as sleep quality [31]. The stress of school work also has been found to cause daytime sleepiness in school children [32]. The daytime sleepiness is found to be an independent predictor of poor academic performance [33] and found to be associated with depression and emotional problems [31, 34]. Daytime sleepiness is also used as an indicator of health status in both clinical and healthy population [35].

Since sleep disturbances and chronic sleep deprivation are prevalent among adolescents worldwide [36], which are closely linked to their functional outcomes, adolescents require programs to improve their sleep health and need education regarding the after effects of sleep restriction or deprivation. Sleep health intervention programs can remain comprehensive to include sleep health education as well as promoting good sleep behaviors [26]. The target group of such programs is healthy adolescents with an irregular sleep schedule, who have less than 8 hours of school night sleep and a discrepant weekday-weekend out of bedtimes. The aim of such programs is to regularize the sleep patterns in adolescents using psychoeducational, motivational, behavior-experimental methods with discussions to maintain healthy sleep behaviors and relapse prevention [37–39]. A previous study has reported the use of a psychoeducational program like “STEPS” to reduce students sleep difficulties and improve sleep habits [40]. In another study, prevention and intervention efforts to promote and enhance sleep behaviors, such as teaching, savoring,or imagery, and removing technology from bedroom are suggested. Also, the chronotherapy principles, with slow, planned shifts towards earlier bedtimes and rise times with dim light conditions prior to bedtime and bright light conditions on waking, are recommended to overcome the delayed sleep phase which is commonly seen in adolescents [20, 41], as well as sleep hygiene with stimulus control instructions [19].

But unfortunately, most of the adolescent sleep promotion programs which were conducted so far focused on enhancing knowledge on sleep [37, 42] and only a few of the studies were found to have resulted in improving the sleep behaviors [40, 43] or sleep problems [44] in adolescents. Some studies have investigated the effect of other interventions on improving adolescents sleep such as use of cognitive-behavior therapy [19] and sleep hygiene intervention programme [39]. However, to the best of our knowledge, none of the studies have focused on interventions for improving adolescent sleep in India. This current study thus aimed to examine changes following a sleep promotion program in sleep hygiene, sleep quality, daytime sleepiness, and present functioning in adolescents. Therefore, we developed a sleep promotion program (SPP), which is a combination of sleep hygiene practice education, video-based visualization (imagery) training, and an educational session with some tips for time management in adolescents. We hypothesized that the SPP would improve adolescents' sleep hygiene practices, sleep quality, and daytime functioning (e.g., daytime sleepiness and emotional and overall distress). The main purpose of the study was to adapt the SPP for use in adolescents studying in various schools in Mangalore, India, and to undertake a pilot trial to determine the efficacy of sleep promotion program and to evaluate the feasibility issues before conducting the study as a randomized controlled trial in a larger sample of adolescents.

## 2. Materials and Methods

### 2.1. Development of the Intervention

The intervention was developed prior to the pilot study, based on the extensive review of the literature. The sleep promotion program consisted of three components and was designed to be delivered in the classroom: a sleep hygiene education session, which is a 50-minute educational session, followed by two sessions of visualization or imagery training, which was done as video-based progressive sessions for stress reduction and relaxation, and a short session consisting of tips for time management. The details of the sessions are given in Table 1. Participants were asked to practise the visualization technique for 5–10 minutes before they sleep every day. Pretesting of the intervention module and the sleep questionnaires was done with 38 adolescents prior to the pilot study. The participants' feedback was taken and they were asked to indicate if any of the components caused a distraction and whether they were able to follow the instructions for relaxation. Based on their feedback, further modifications on the intervention module were done.

### 2.2. Design

The study used a two-arm, parallel, randomized controlled pilot trial aimed at investigating the effectiveness and feasibility of the SPP as a part of doctoral study research. The school for the intervention and the school participating as control were selected through tossing the coin. The ethical permission was obtained from the Central Ethical Committee of the University, dated 16/04/2012 (NU/CEC/Ph.D-44/2011). The study was conducted by random selection of the schools. In addition, permission was obtained from the school Principal, or Head of the school, and the participants were required to give their assent and their parents were required to give their consent for the study.

Participants' demographic characteristics with some additional questions on sleep and activities, along with standardized questionnaires for sleep, were used for measuring sleep outcomes. The outcomes were measured at three time points using self-report questionnaires: before program, then after 14 days of intervention, and after 6 weeks of intervention. The main outcome measures were sleep hygiene, sleep quality, daytime sleepiness, and adolescents' emotional and overall distress.

### 2.3. Participants

Participants were 58 healthy adolescents, aged between 11 and 17 years, and consisted of 29 males and 29 females studying from 6th class to 12th class. The mean age of the adolescents was 14.02 ± 2.15 years. They were included if they can comprehend English language, and if they were currently studying in various schools in Mangalore, India. They were excluded if they had any chronic medical or psychiatric problems, or if they were currently on psychotherapy, counseling, or treatment for any sleep problems and allergic disorders. The consent forms were distributed in the class and collected two days before the commencement of the study. The participants were selected through stratified random sampling method. The stratification was based on gender and grade levels of the adolescents.

**Figure 1:**
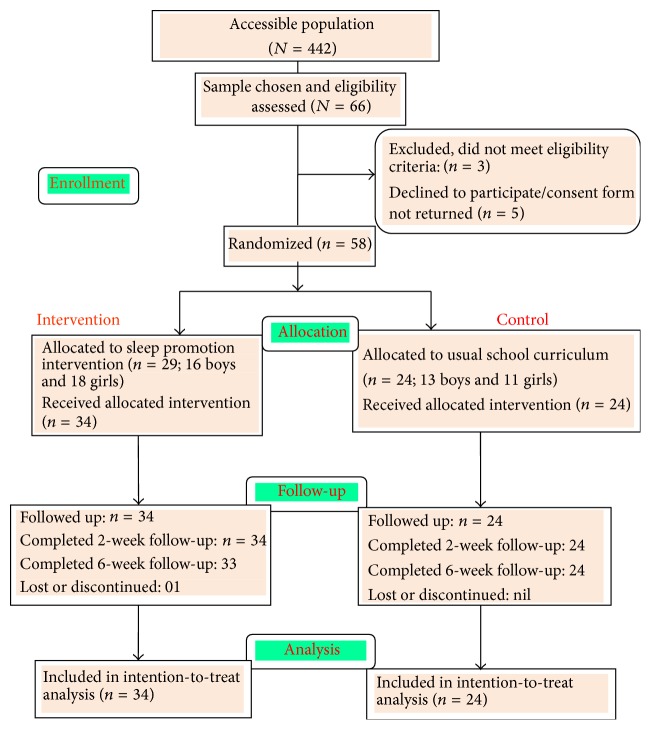
Progression of participants in the flow diagram.

Participants in the intervention group received the full SPP intervention, and the control group continued with their school curriculum, and their usual health and physical education.  Figure 1 shows the progression of the participants in the flow diagram.

### 2.4. Instruments and Tools

#### 2.4.1. Sleep Questionnaire

The sleep questionnaire consisted of sociodemographic information, with few supplementary questions on items of sleep and activity (e.g., extra coaching classes or tuitions, average sleep duration on weekdays and weekends, any problems at home or other factors affecting sleep, and engagement in the activities other than studies such as sports, music, games, and so forth). The sleep duration was measured by questions like “How much sleep do you get on an average during a school day?” and “How much sleep do you get on an average during a weekend?” and the participants were asked to indicate it in hours/per day. The questionnaire was validated by sending it to a team of subject experts. The total items in the questionnaire were 16.

#### 2.4.2. Sleep Hygiene Index (SHI)

Developed by Mastin et al. [45], the SHI is a 13-item questionnaire on a five-point Likert scale (*never: 1*;* rarely: 2*;* sometimes: 3*;* frequently: 4*;* always: 5*) to assess the presence of sleep behaviors constituting sleep hygiene. Item scores summed provide a global assessment of sleep hygiene. The scores range from 13 to 65, with higher scores indicating a more maladaptive sleep hygiene status. The original scale items number 4, “I use alcohol, tobacco, or caffeine within four hours of going to bed or after going to bed,” item 9, “I use my bed for things other than sleeping or sex,” and item 12, “I do important work before bedtime (e.g., pay bills, schedule, or study),” were slightly modified to suit the adolescents in the present study. For example, the words “alcohol and tobacco” in item 4 and the word “sex” in item number 9 were removed. The words “paying bills” in item 12 were amended to “household chores” [46]. Permission was granted for using the scale and to do the needed modifications by the developers. An acceptable internal consistency coefficient was obtained during the pretesting phase (*α* = 0.708), with a good test-retest reliability (*r* = 0.731).

#### 2.4.3. Pittsburgh Sleep Quality Index (PSQI)

Copyrighted by Buysse et al. [47], this standardized tool is intended to assess the sleep quality and sleep disturbance over a period of one month in both clinical and nonclinical populations. The sleep quality tool consists of seven domain scores like subjective sleep quality, sleep latency, sleep duration, sleep efficiency, sleep disturbance, sleep medication, and daytime dysfunction, and the component scores summed up produce a global score which ranges from 0 to 21, with scores greater than “5” indicating worse sleep quality. The scoring of component 3, which is the “sleep duration,” was slightly modified to suit the adolescents' recommended optimal sleep duration and the responses were made as “>9 hours = 0, 8.5–9 hours = 1, 8–8.5 hours = 2, and <8 hours = 3.” Permission was obtained for using the scale and for altering this item from the author. The sensitivity of the tool is 89.6% and specificity is 86.5% in distinguishing “good” and “poor sleepers.” PSQI has good reliability with high internal consistency (*α* = 0.83) and a good test-retest reliability (*r* = 0.85).

#### 2.4.4. Cleveland Adolescent Sleepiness Questionnaire (CASQ)

Copyrighted by Spilsbury et al. [48], this instrument contains 16 items to measure the daytime sleepiness in adolescents. The scores range from 16 to 80 on a 5-point Likert scale (never: 1; rarely: 2; sometimes: 3; often: 4; almost every day: 5). Five of the statements are negatively scored. The daytime sleepiness is obtained by summing up the total of 16 items' scores, and the higher the score is, the higher the daytime sleepiness is. The scale has a high internal consistency, Cronbach's alpha = 0.89, and can be used both in clinical (e.g., those who have obstructive sleep apnea or OSA) and in nonclinical healthy normal sample of adolescents. Permission was obtained for using this scale in our study.

#### 2.4.5. PedsQL*™* Present Functioning Visual Analogue Scale (PedsQL*™* VAS)

Copyrighted by James W. Varni, 1998 [49], The PedsQL*™* VAS instrument is a visual analogue scale containing six items that measure anxiety, sadness, anger, worry, fatigue, and pain at the present moment. The Total Symptom Score is calculated by taking the average of all six items, while the Emotional Distress Summary Score represents the mean of the anxiety, sadness, anger, and worry items on a 0–100 mm scale. The test-retest reliability of the scale for the total score is 0.85 (for child and teen self-report) and for the emotional distress summary is 0.78.

#### 2.4.6. Power Analysis

As this was a pilot study, power analysis was not performed, and we focused on the reliability of the instruments and tools used so that it would assist in carrying out a RCT on a large scale. With reference to a previous research conducted as RCT [39], 22 participants are sufficient to provide 80% power when using a two-sided test at 0.05 level for continuous measures, and a sample size of 28 participants was adequate in the same way for showing the reliability of categorical measures. Our final sample size was 58 adolescents in this pilot study.

## 3. Results

### 3.1. Demographic Characteristics and Mean Sleep Duration

**Figure 2:**
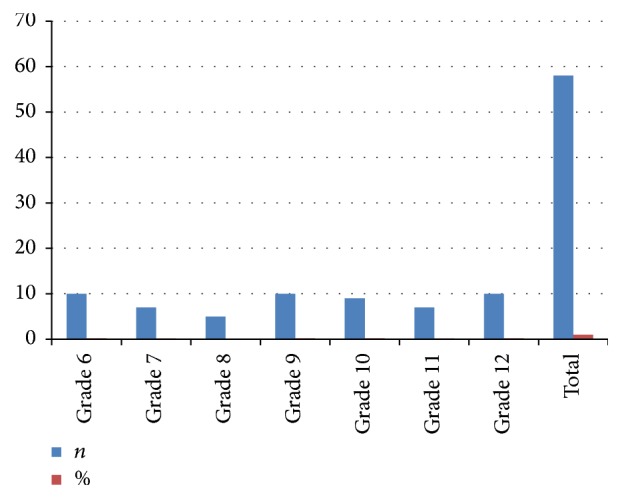
Grade level distribution of the adolescent participants.

Fifty-eight adolescents (29 males and 29 females) in the age group of 11–17 years participated in the study. Thirty-four adolescents (58.6%) were in the experimental group and 24 adolescents (41.4%) were in the control group respectively. The mean age of the adolescents was 14.02 ± 2.15 years. The grade level distribution of the adolescent participants is shown in Figure 2.

The adolescents obtained an average of 7.68 ± 0.99 h of sleep on school days, and on weekends they obtained a slightly longer sleep duration, that is, 8.70 ± 1.63 h of sleep, with total sleep duration of 8.19 ± 1.12 at baseline. Sleep duration showed a significant improvement at 2 weeks (*M* = 9.06 ± 5.68, *p* < 0.05), whereas it was not significant at 6 weeks (*M* = 8.11 ± 1.47, *p* > 0.05). The sleep duration was significantly correlated with the sleep quality (*p* < 0.05) and negatively correlated with daytime time sleepiness (*r* = −0.295, *p* value 0.025 at <0.05). The sleep duration was also found to significantly decrease with increasing age level of the participants on both weekdays and weekends (*p* value < 0.01).

There were no significant differences among any of the sleep variables as per gender (*p* value > 0.05 level of significance).

### 3.2. Changes in the Outcome Measures at Baseline and at 2-Week and 6-Week Follow-Up

The average sleep hygiene scores obtained at baseline were 28.59 ± 6.71. Sleep hygiene practices measured using ANCOVA did not show any significant differences between the experimental and control groups after the intervention (*p* > 0.05) at various time points. But a significant correlation of sleep hygiene with sleep quality, daytime sleepiness, and emotional and overall distress was observed in the experimental and the control groups (*p* < 0.01) of adolescents.

The average sleep quality scores of adolescents at baseline were 6.4 ± 2.4. A significant difference between the experimental and control groups after the intervention measured by Mann-Whitney *U* test was observed in the sleep onset latency, sleep duration, and overall sleep quality among adolescents (*p* < 0.01), whereas subjective sleep quality, sleep efficiency, sleep disturbance, and daytime dysfunction did not show any significant improvement after the intervention (*p* > 0.05). Sleep quality was significantly associated with sleep hygiene scores (*t* = 4.9, *p* = 0.000, <0.01) and showed a positive correlation with daytime sleepiness scores (*r* = 0.317, *p* = 0.015, at <0.05 level of significance).

The mean daytime sleepiness scores among adolescents were 36.83 ± 8.3 at baseline. A significant difference between the experimental and control groups in daytime sleepiness measured using ANCOVA was observed after the intervention only at 6 weeks (*F* = 5.68, *p* = 0.021, at <0.05), whereas it was not significant at 2 weeks (*p* > 0.05).

The mean emotional distress scores of adolescents at preprogram level were 22.9 ± 17.6 and the overall distress scores were 23.2 ± 16.1. Forty-five adolescents (77.6%) showed mild emotional and overall distress. The emotional distress and overall distress showed a significant improvement in the experimental group in comparison with the control group measured using ANCOVA at 2 weeks (*F* = 4.51, *p* = 0.038, at <0.05, and *F* = 4.28, *p* = 0.043, at <0.05, resp.), but it was not significant at 6 weeks (*p* > 0.05). The emotional and overall distress scores was significantly correlated with sleep quality (*r* = 0.368, *p* = 0.032, at <0.05).

### 3.3. Program Acceptability

The program attendance was very high (96.55% attended all sessions, and one participant missed one session), and 28 out of 29 participants in the intervention group participated in the exit survey. Nearly all participants stated that the questions were clear (98.27%) and the intervention was helpful (100%). However, few of the participants felt that answering the questionnaire was slightly boring (5.17%) and a part of the visualization video was slightly distracting (6.89%).

## 4. Discussion

The current study was aimed at determining the changes following a newly developed sleep promotion intervention on sleep hygiene practices, sleep quality and daytime sleepiness, and present functioning among healthy adolescents aged 11–17 years in a pilot trial, to see the feasibility issues and applicability of this study in adolescents prior to a larger randomized controlled trial.

The adolescents in our study showed a significant difference between the weekdays and weekends sleep, with an increase in weekend sleep duration as a compensatory mechanism of lost sleep, and it was found to increase with increasing age. Similar results were reported in studies conducted in Italy [6], Australia [19], and Japan [10].

The study also showed a positive effect on adolescent's sleep duration at 2 weeks and daytime sleepiness at 6 weeks. A few studies had also reported an increase in the total sleep time [44] and improvement in daytime sleepiness [39] after the intervention. No significant effect was observed in the sleep hygiene scores in our study, in contrast with studies conducted in New Zealand [39] and the US [40].

Moreover, significant improvements in sleep onset latency, overall sleep quality, and sleep duration were noticed in our study. In two other experimental studies, significant improvement in the sleep onset latency was observed in the treatment group [40, 44]. Whereas, in another Australian based randomized controlled trial study, there was not an impact on sleep intervention on different sleep parameters like total sleep duration, daytime sleepiness, and depressed mood, apart from regularization of the out of bed times, and the effect disappeared at 6 weeks of follow-up [19]. Daytime sleepiness in our study was negatively correlated with the total night time sleep, similar to other studies [22, 34].

However, our pilot study, which was a feasibility study, has some limitations. Being a pilot study, the sample size of the participants was very small, and they were recruited from two schools, which are not highly representative of the population. Second, the sleep parameters were assessed by self-reported questionnaires. Future studies could consider larger number of participants representing a wider geographic area to get the best results. As for sustained results, a trial with a longer duration and the commitment from the part of the adolescents is required, since they are tightly bound with the academic commitments. However, despite these limitations, this intervention program holds promise for healthy adolescents to improve their sleep behaviors, and the effect of the sleep promotion program treatment has yet to be proven through a future research.

## Figures and Tables

**Table 1:** The sleep promotion program contents of the session for adolescents.

Session number	Duration	Content
1	30 minutes	Definition of sleep
		Functions of sleep
		Various sleep stages: REM and NREM sleep
		Factors causing sleep deprivation in adolescents
		The impact of sleep problems in adolescents: physical, cognitive, emotional and social, and motivational

2	20 minutes	Definition of sleep hygiene practices
		The importance of sleep hygiene practices in adolescents
		(i) Sleep complaints are common in adolescents
		(ii) Circadian process and sleep-wake homeostasis of sleep regulation
		(iii) Natural sleep shift in adolescents
		(iv) The relation between sleep and memory
		How to establish good sleep hygiene practices

3	25 minutes	Visualization training: day 1 (a video-based training using imagery for stress reduction and relaxation)

4	25–30 minutes	Visualization training: day 2 (a video-based training for stress reduction and relaxation)

5	15 minutes (session combined with second day of visualization)	Tips for time management skills
